# Bovine tongue epithelium-derived cells: A new source of bovine mesenchymal stem cells

**DOI:** 10.1042/BSR20181829

**Published:** 2020-04-17

**Authors:** Jienny Lee, Jeong Su Byeon, Na-Yeon Gu, Siu Lee, Se-A Lee, Da-Un Jeong, In-Ohk Ouh, In-Soo Cho, Jae-Young Song, Yoon-Hee Lee, Bang-Hun Hyun

**Affiliations:** Viral Disease Research Division, Animal and Plant Quarantine Agency, 177, Hyeoksin 8-ro, Gimcheon-si, Gyeongsangbuk-do 39660, Republic of Korea

**Keywords:** Bovine, Mesenchymal stem cells, Tongue tissue

## Abstract

Mesenchymal stem cells (MSCs) possess the ability to differentiate into multiple cell lineages, and thus, confer great potential for use in regenerative medicine and biotechnology. In the present study, we attempted to isolate and characterize bovine tongue tissue epithelium-derived MSCs (boT-MSCs) and investigate the culture conditions required for long-term culturing of boT-MSCs. boT-MSCs were successfully isolated by the collagenase digestion method and their proliferative capacity was maintained for up to 20 or more passages. We observed a significant increase in the proliferation of boT-MSCs during the 20 consecutive passages under low-glucose Dulbecco’s modified Eagle’s medium culture condition among the three culture conditions. These boT-MSCs presented pluripotency markers (octamer-binding transcription factor 3/4 (Oct3/4) and sex determining region Y-box2 (Sox2)) and cell surface markers, which included CD13, CD29, CD44, CD73, CD90, CD105, CD166, and major histocompatibility complex (MHC) class I (MHC-I) but not CD11b, CD14, CD31, CD34, CD45, CD80, CD86, CD106, CD117, and MHC-II at third passage. Moreover, these boT-MSCs could differentiate into mesodermal (adipocyte, osteocyte, and chondrocyte) cell lineages. Thus, the present study suggests that the tongue of bovines could be used as a source of bovine MSCs.

## Introduction

Mesenchymal stem cells (MSCs) are multipotent cells with the ability to differentiate into several cell lineages, and thus hold therapeutic implications for cell therapy in field, such as regenerative medicine and reproductive biotechnology. Although MSCs have been isolated from many species, which includes humans and animals, data are currently limited on isolation from large animals, such as cattle and sheep, using their ruminants. Bovine is an important agricultural species with significant commercial value and an attractive large animal model for biomedical and biotechnology research. The development of large animal experimental models, which includes cattle, may provide alternative strategies to investigate MSCs physiology and potential application in human and veterinary regenerative medicine [1].

Mammalian tongue is an important digestive and sensory organ that has multiple functions, such as food intake, taste and touch sensation, and a biolinguistic role as an articulatory organ. The surface of the tongue is covered with stratified squamous epithelial cell layers. Lingual dorsal epithelium contains four different kinds of papillae: filiform, fungiform, foliate, and circumvallate papillae. Stratum corneum is observed in filiform papillae, but not in fungiform, foliate, or circumvallate papillae. In contrast, taste buds are observed in fungiform, foliate, and circumvallate papillae, but not in filiform papillae. The lingual epithelium is renewed continually throughout the life of mammals. The turnover rate of the mouse lingual epithelium, which is at a rate of 6–7 days, is four- to five-fold higher than that of dorsal skin [2], which suggests the existence of stem cells in the papillae. The stem cell niche for lingual epithelial stem cells is unknown. Hume and Potten [3] revealed that mouse lingual epithelial stem cells are located in the basal layer of the lingual epithelium – similar to other epithelial tissues that employ the 3H-TdR label-retaining assay. Several research groups have proposed candidates for lingual epithelial stem cells [4]. However, the stem cell markers that were used in their studies were not specific to stem cells based on their detection in a portion of the mature epithelial cells. Actual stem cells that are responsible for the long-term maintenance of lingual epithelium have not yet been identified.

All organs develop and consist of an epithelium and mesenchyme that share common morphological features during the early stages of morphogenesis. In certain interactions, epithelium is able to induce differentiation of the mesenchyme and *vice versa*, and plays an instructive role that is mediated by the differential activation of genes in responding epithelial cells. Epithelial–mesenchymal interactions have been described in detail by experimental embryologists as early as in the 1950s and 1960s [5]. Many researchers have sought to establish bovine MSCs from various tissues. To date, bovine MSCs have been isolated from bone marrow [6], umbilical cord blood [7], amniotic fluid [1], liver [8], adipose tissue [9], endometrial tissue [10], and Wharton’s jelly [11]. The objective of the present study was to isolate MSCs from bovine tongue tissues, and to fully characterize bovine tongue tissue epithelium-derived MSCs (boT-MSCs) by analyzing cell growth curves, cell surface markers, and stemness such as the mesodermal differentiation potential of Korean native cattle.

## Materials and methods

### Isolation and culture of boT-MSCs

boT-MSCs were isolated from each tissue according to a general method that has been described in previous studies [12–15] with minor modifications. First, bovine tongue tissues were prepared from 30-month-old Korean native cattle (*n*=3). Bovine tongue epithelium from the dorsum of the bovine tongue was removed and isolated for further procedure. Blood vessels and muscles were removed with sterile scissors and the forceps that remained were the stratified squamous epithelium. These tissues were minced into 1–2 mm pieces and incubated in phosphate-buffered saline (PBS, Gibco, CA) that contains 0.1% collagenase type Ι at 37°C for 1 h. Digested tissues were filtered with a 100-μm cell strainer (Becton Dickinson, U.S.A.) and centrifuged at 1500 rpm for 10 min. Subsequently, cell pellets were resuspended in three different media (low-glucose Dulbecco’s modified Eagle’s medium (DMEM), low-glucose DMEM and Ham’s F12 medium at 1:1 [12] and Iscove’s DMEM (IMDM) and Ham’s F12 medium at 1:1 [13,14]) that were supplemented with 1% penicillin–streptomycin (Gibco, U.S.A.) and 10% fetal bovine serum (FBS, Gibco, U.S.A.). Cells were seeded at a density of 1 × 10^6^ cells in a T175 flask and maintained in a humidified incubator at 5% CO_2_ and 37°C. After 2 days, the cell culture medium was refreshed and the cells were passaged every 4 days by trypsinization upon reaching 80% confluence. Cells were used for subsequent analyses at a third passage (P3) or fourth (P4). Between each subpassage, cellular viability was measured using the Trypan Blue exclusion assay.

### Fibroblastic colony-forming unit assay

The fibroblastic colony-forming unit assay (CFU-F) assay was constructed with cells on P3 according to Mensing et al. [16] with minor modifications [15]. For this, cells were seeded (100 cells/well) into six-well plates and cultured in a low-glucose DMEM medium for 5 days. These cells were then washed with PBS twice. After fixing with 4% paraformaldehyde for 10 min at room temperature, cells were then washed with PBS. Next, cells were stained with Crystal Violet in 100% methanol to visualize the colony, washed with PBS, and allowed to dry. Stained cells were visualized under an inverted microscope (40×).

### Calculating cumulative population doubling level and cell doubling time

During continuous passages, cells were seeded at a density of 5 × 10^4^ cells/well in six-well culture plates (*n*=3) and subcultured for 4 days with medium that was refreshed every 2–3 days [17]. The number of cells at the time of both seeding and harvesting were determined to calculate cumulative population doubling level (CPDL) based on the following formula: CPDL = ln(N_f_/N_i_)/ln2 (N_i_, the initial cell number; N_f_: the harvest cell number). The cumulative doubling level was obtained by adding the doubling level of each passage to that of the previous passage. The cell doubling time (DT) was calculated from the CPDL and cell culture time (CT) for each passage by the following formula: DT = CT/CPDL.

### Flow cytometric analysis

Cells grown on culture plates that were digested with 0.25% trypsin/EDTA and washed with PBS. Cell were washed with cell staining buffer (Biolegend, U.S.A.) prior to staining. To identify stem cell surface markers, cells (5 × 10^5^) were stained with mouse anti-bovine CD29 (Kingfisher Biotech, WS0577B, U.S.A.), phycoerythrin (PE)–conjugated mouse anti-bovine CD44 (AbD serotec, MCA2433PE, U.K.), Fluorescein isothiocyanate (FITC)–conjugated mouse anti-bovine CD45 (AbD serotec, MCA832F, U.K.), FITC–conjugated mouse anti-human CD90 (Novusbio, NBP2-47755F, U.S.A.), mouse anti-bovine major histocompatibility complex (MHC) class I (MHC-I; Kingfisher Biotech, WS0558B, U.S.A.), PE–conjugated mouse anti-bovine MHC-II (Mybiosource, MBS224588, U.S.A.), FITC–conjugated mouse anti-bovine CD11b (Bio-Rad, MCA1425F, U.K.), FITC–conjugated mouse anti-bovine CD80 (Bio-Rad, MCA2436F, U.K.), and FITC–conjugated mouse anti-bovine CD86 (Bio-rad, MCA2437F, UK) for 30 min at 4°C. Unconjugated antibodies (CD29 and MHC-I) were treated with anti-mouse IgG FITC secondary antibodies for 30 min. Cells were then washed with PBS twice. Isotype controls were run in parallel as negative controls. A minimum of 10000 cells were analyzed. Flow cytometry analyses were performed using an FACS Calibur™ flow cytometer (Becton Dickinson, U.S.A.) with Cell Quest Pro software (Becton Dickinson, U.S.A.) for data analysis.

### Reverse transcriptase-polymerase chain reaction and quantitative real-time RT-PCR

Total RNAs were extracted using an RNeasy Mini kit (Qiagen, U.S.A.) according to the manufacturer’s instructions. The RNA concentration was determined by measuring the absorbance at 260 nm with a spectrophotometer (Thermo, U.S.A.), and cDNA were generated using total RNA (2 μg), reverse primers (10 pmol of each), and GoScript™ Reverse Transcriptase (Promega, U.S.A.). Real-time PCR analysis was carried out in 96-well plates with a LightCycler® 480 SYBR Green I Master Mix (Roche Diagnostics, U.S.A.). The following program was used for amplification: predenaturation for 10 min at 95°C, followed by 45 cycles of denaturation for 10 s at 95°C, annealing for 10 s at 60°C, and elongation for 10 s at 72°C. The fold difference in the gene expression in differentiated MSCs compared with that in undifferentiated MSCs was calculated using the 2^−ΔΔ*C*_t_^ method as described by Livak and Schmittgen [18]. Glyceraldehyde-3-phosphate dehydrogenase (GAPDH) was used in every quantitative real-time RT-PCR (qRT-PCR) analysis as an internal control and reference. Expression of this gene was unchanged between treatments [19]. Primer sequences and their respective annealing temperatures are presented in Table 1.

**Table 1:** List of primers used for reverse transcription-polymerase chain reaction

Gene	Primer sequence (5′–3′)	PCR product size (bp)	Annealing temperature (°C)	Accession number
*CD13*	F-CCC ACC TGG AAT CTG AAA GA	92	60	NM_001075114.1
	R-GTG GTC AGT GGG TGA GAG GT			
*CD14*	F-GCA GCC TGG AAC AGT TTC TC	178	60	NM_174008.1
	R-TCC TCA AGC GTC AGT TCC TT			
*CD31*	F-TCT GTT TGC CTT TGC TCC TT	113	60	NM_174571.3
	R-GCA GGA GAG GTC ATG GAG AG			
*CD34*	F-CAT GCC GTC TTA ACC CAT CT	139	60	NM_174009.1
	R-CGG TCT ACA GAG GTG GTG GT			
*CD29*	F-TGT CGA GTG TGT GAG TGC AA	193	60	NM_174368
	R-AGA CTC CAA GGC AGG TCT GA			
*CD44*	F-CCG GAA CAT AGG GTT TGA GA	160	60	NM_174013
	R-TGA GGC ATT GAA GCA GTA CG			
*CD45*	F-CCA CGG GTA TTC AGC AAG TT	244	52	NM_001206523
	R-CCC AGA TCA TCC TCC AGA AA			
*CD73*	F-GTG TCG TGT GCC CAG TTA TG	90	60	NM_174129.3
	R-AAT CCG TCT CCA CCA CTG AC			
*CD90*	F-GTG AAC CAG AGC CTT CGT CT	201	60	NM_001034765
	R-GGT GGT GAA GTT GGA CAG GT			
*CD105*	F-CTG ATC CTC AGC GTG AAC AA	226	60	NM_001076397
	R-GAC GAA GGA AGA TGC TTT GC			
*CD106*	F-CAG GCT GTG AGT CTC CAT CA	178	60	BC151459.1
	R-TGG ATT GCT TTC TCC AGC TT			
*CD117*	F-ACT CCC TGT GAA GTG GAT GG	119	60	AF263827.1
	R-AGG GGC TGC TTC CTA AAG AG			
*CD166*	F-GAT GTG AAA CGC AAT GCA AC	85	60	NM_174238.1
	R-GAA CTG TGA TGG CTG CTG AA			
*MHC-II*	F-AGC CTC TGT GGA GGT GAA GA	157	60	NM_001013601
	R-GCT GCC AGA CAG TCT CCT TC			
*Sox2*	F-CAC AAC TCG GAG ATC AGC AA	162	60	BC133458
	R-CAT GAG CGT CTT GGT TTT CC			
*Oct3/4*	F-GTT TTG AGG CTT TGC AGC TC	182	55	NM_174580
	R-CTC CAG GTT GCC TCT CAC TC			
*C/EBPα*	F-ATC GAC ATC AGC GCC TAC AT	138	60	NM_176784
	R-CGG GTA GTC AAA GTC GTT GC			
*PPARγ*	F-CAG TGT CTG CAA GGA CCT CA	128	60	NM_181024
	R-GAT GTC AAA GGC ATG GGA GT			
*LPL*	F-TGC TGG TAT TGC AGG AAG TC	124	60	NM_001075120
	R-AAA ATC CGC ATC ATC AGG AG			
*Collagen type II*	F-CTC AAG TCC CTC AAC AAC CAG	134	60	NM_001113224
	R-TTG GGG TCG ATC CAG TAG TC			
*Aggrecan*	F-CAG TCA CAC CTG AGC AGC AT	104	60	NM_173981
	R-CCT TCG ATG GTC TTG TCG TT			
*Sox9*	F-AGA AGG ACC ACC CGG ACT AC	134	60	XM_024981096
	R-CGT TCT TCA CCG ACT TCC TC			
*Osteocalcin*	F-TGA CAG ACA CAC CAT GAG AAC CC	320	60	X53699
	R-AGC TCT AGA CTG GGC CGT AGA AG			
*Collagen type I*	F-TGC TGG CCA ACC ATG CCT CT	120	60	AB008683
	R-CGA CAT CAT TGG ATC CTT GCA G			
*GAPDH*	F-CCT TCA TTG ACC TTC ACT ACA TGG TCT A	127	60	U85042
	R-TGG AAG ATG GTG ATG GCC TTT CCA TTG			

### *In vitro* differentiation

For adipogenic and osteogenic differentiation, cells were seeded on to six-well plates that contain differentiation medium. The composition of the differentiation medium is shown in Table 2. For chondrogenic differentiation, cells were cultured in 5 μl droplets of growth medium in four-well plates for 3 h in the presence of 5% CO_2_ and changed with chondrogenic differentiation medium plus transforming growth factor β-3 (TGF-β3; Lonza, U.S.A.). All differentiation media were changed every 2–3 days and the differentiation to the three cell lineages was evaluated after 21 days.

**Table 2:** Composition of the differentiation medium

Adipogenesis		Osteogenesis		Chondrogenesis	
Dexamethasone (Sigma, D1756)	1 µM	Dexamethasone	0.1 µM	Dexamethasone	0.1 µM
Indomethacin (Sigma, I7378)	500 µM	β-glycerophosphate (Sigma, G9422)	10 mM	l-ascorbic acid 2-phosphate	50 µg/ml
Insulin (Sigma, I6634)	20 µM	l-ascorbic acid 2-phosphate (Sigma, A8960)	200 µM	TGF-β3 (Lonza, PT-4124)	10 ng/ml
3-Isobutyl-1-methylxanthine (Sigma, I5879)	500 µM				

To evaluate the differentiation abilities, cells were washed twice with PBS, fixed with 4% paraformaldehyde for 10 min at room temperature, and then washed with PBS again. For adipogenic differentiation, accumulation of red lipid vacuoles was observed after Oil Red O staining (IHC World, U.S.A.). For osteogenic differentiation, extracellular calcium deposition was confirmed by Alizarin Red staining (IHC World, U.S.A.). For chondrogenic differentiation, the presence of glycosaminoglycan was verified by Alcian Blue staining (IHC World, U.S.A.). Stained cells were visualized using an inverted microscope.

### Statistical analysis

The relative expression of differentiation marker genes was analyzed by one-way analysis of variance (ANOVA). Differences between the two methods were compared by a Student’s *t* test (JMP® 6.0; SAS Institute Inc., Cary, NC, U.S.A.). *P*-value less than 0.05 were considered statistically significant.

## Results

### Isolation and characterization of boT-MSCs

**Figure 1:**
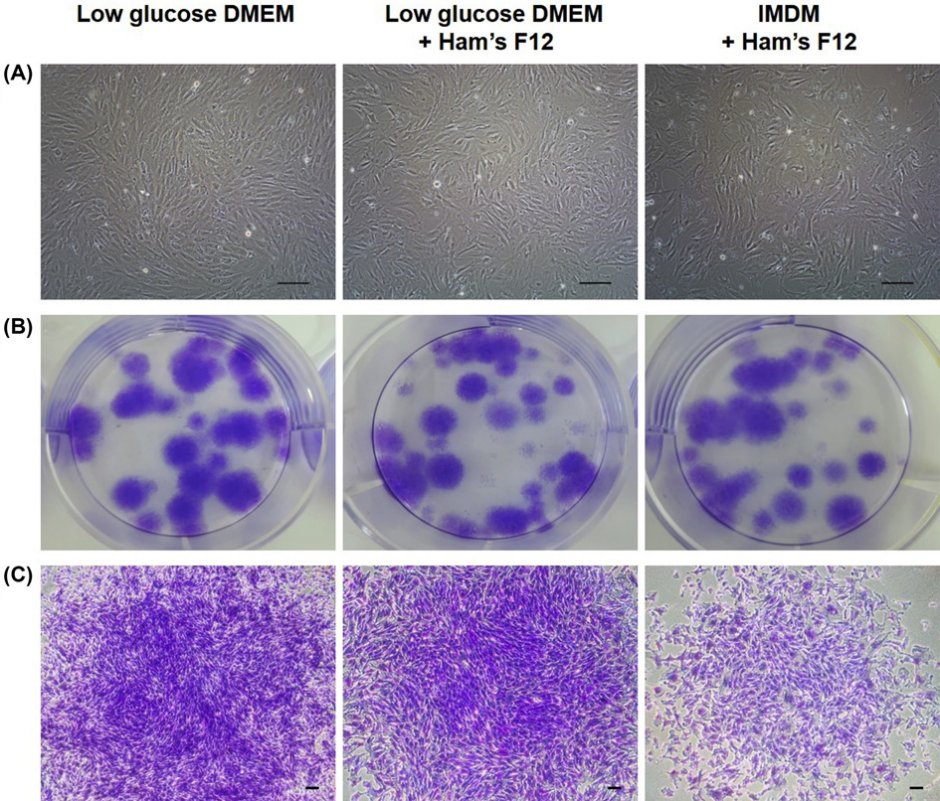
Morphology of boT-MSCs (**A**) Fibroblast-like morphology of cells (P2, day 4) from boT-MSCs that were cultured in low-glucose DMEM, low-glucose DMEM/Ham’s F12, and IMDM/Ham’s F12; 100× (scale bars = 100 μm). (**B**) Photomicrographs of cells stained with Crystal Violet displaying colony-forming capacity. (**C**) CFU-F (P3, day 5) capacity and microscopic images of boT-MSCs that were cultured in low-glucose DMEM, low-glucose DMEM/Ham’s F12, and IMDM/Ham’s F12; 40× (scale bars = 100 μm).

**Figure 2:**
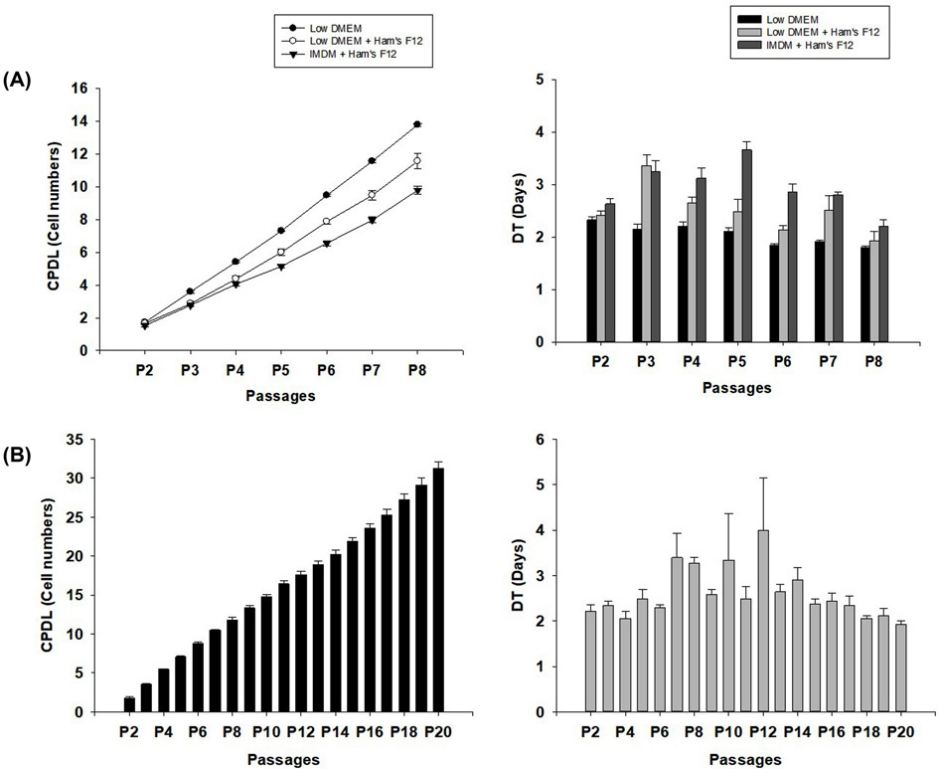
CPDL and cell DT of boT-MSCs (**A**) CPDL of boT-MSCs increased at each passage, and the DT was evaluated until P8 when the cells were cultured in three different mediums: low-glucose DMEM, low-glucose DMEM/Ham’s F12, and IMDM/Ham’s F12. (**B**) CPDL and DT of boT-MSCs that were cultured in low-glucose DMEM were examined at each passage, until P20.

Cells obtained from the bovine tongue epithelium exhibited the ability to attach to culture plates and expand *in vitro* (Figure 1A). The cells also demonstrated tremendous capacity of CFU-F at P3 (Figure 1B,C). To test the culture conditions for bovine tongue epithelium-derived cells, various media of the cells were tested *in vitro* by examining P8 (Figure 2A). Among the three different examined culture conditions (low-glucose DMEM, low-glucose DMEM/Ham’s F12, and IMDM/Ham’s F12) that were supplemented with 1% penicillin–streptomycin (Gibco, U.S.A.), and 10% fetal bovine serum (FBS, Gibco, U.S.A.), cells cultured in the low-glucose DMEM medium demonstrated the highest CPDL during eight consecutive passages. The cells were further cultured in low-glucose DMEM. Consequently, a continuous increase in the CPDL and constant DT of cells for 20 or fewer passages was observed (Figure 2B).

**Figure 3:**
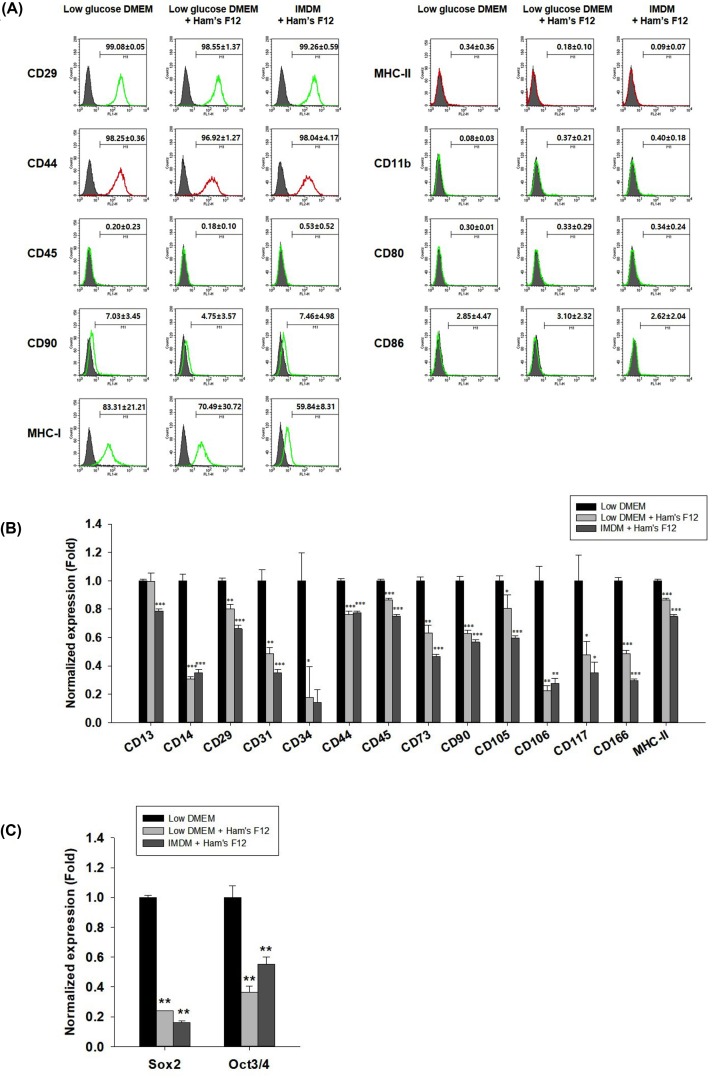
Cell surface markers of boT-MSCs (**A**) Cell surface markers (positive: CD29, CD44, CD90, and MHC-I; negative: CD45, CD11b, CD80, CD86, and MHC-II) of boT-MSCs were observed (P3) by flow cytometry analysis. Data are expressed as means ± standard error of values, and were obtained by three determinations. (**B**) Cell surface markers (CD13, CD14, CD29, CD31, CD34, CD44, CD45, CD73, CD90, CD105, CD106, CD116, and MHC-II), and (**C**) pluripotency markers (Sox2 and Oct3/4) of boT-MSCs were observed (P3) by qRT-PCR. GAPDH was used as a housekeeping control gene. Results are shown as means ± standard error (*n*=3) (*P<0.05, **P<0.001 and ***P<0.0001).

To characterize the MSCs, cell surface markers were analyzed by flow cytometric analysis and qRT-PCR analysis at P3. Results of the flow cytometry and qRT-PCR at P3 indicate that the cells were strongly positive for CD13, CD29, CD44, CD73, CD90, CD105, CD166, and MHC-I, but negative for CD11b, CD14, CD31, CD34, CD45, CD80, CD86, CD106, CD117, and MHC-II (Figure 3A,B). In addition, bovine tongue epithelium-derived cells evidently expressed stemness markers such as sex determining region Y-box2 (Sox2) and octamer-binding transcription factor 3/4 (Oct3/4). At P3, the expression levels of stemness markers (Sox2 and Oct3/4) were higher for cells that were grown in the low-glucose DMEM medium compared with those that were grown in the other medium (Figure 3C).

### Differentiation potentials of boT-MSCs

**Figure 4:**
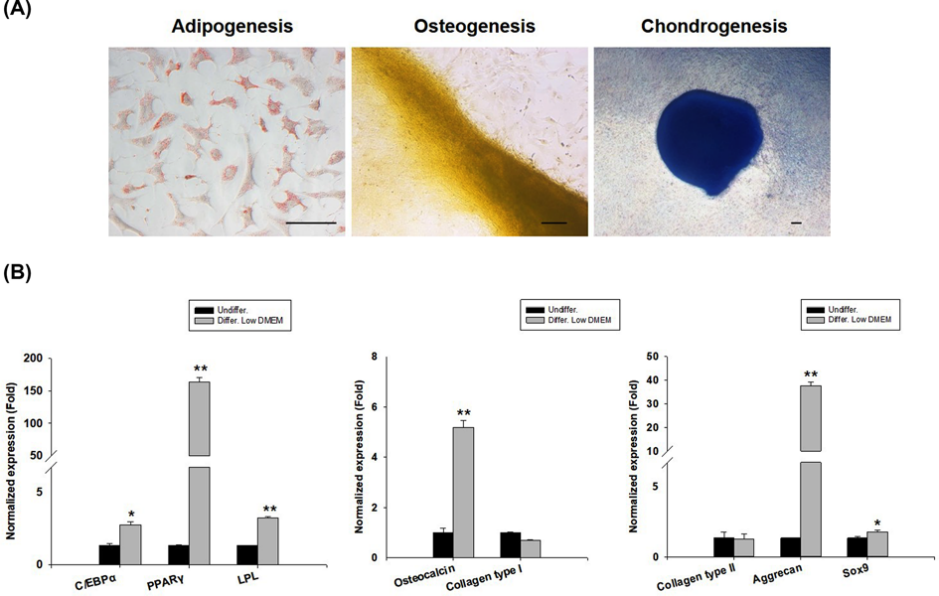
Differentiation potentials of bovine tongue tissue-derived MSCs (**A**) Adipocytes (Oil Red O, 200×), osteocytes (Alizarin Red, 100×), and chondrocytes (Alcian Blue, 40×) were positively stained after culturing in a low-glucose DMEM at P3 (scale bars = 100 μm). (**B**) mRNA expression levels of adipocyte-, osteocyte-, and chondrocyte-related genes were detected by qRT-PCR and compared between undifferentiated and differentiated cells in the two groups at P3. GAPDH was used as a housekeeping control gene. All mRNA data were normalized to levels of undifferentiated cells. Relative fold changes in the expression levels are shown as means ± standard error (*n*=3) (*P<0.05 and **P<0.001).

To investigate the mesodermal differentiation potentials of bovine tongue epithelium-derived cells, cells were differentiated into adipocytes, osteocytes, and chondrocytes under specific conditions. As a result of adipogenic differentiation, the presence of neutral lipid accumulation was evident in differentiated cells (as indicated by the red color). In addition, the presence of extracellular calcium was confirmed by Alizarin Red staining in differentiated cells, which demonstrated osteogenic potential. Further, deep blue staining of the proteoglycan in differentiated groups was observed during chondrogenic differentiation (Figure 4A).

These results are further confirmed by qRT-PCR analysis. The expression levels of chondrogenic differentiation potential-related genes – such as *aggrecan* and *Sox9* – in bovine tongue epithelium-derived cells were found to be significantly higher than those of undifferentiated cells. Moreover, the expressions levels of the adipogenic and osteogenic differentiation potential-related genes – such as CCAAT/enhancer-binding protein α (C/EBPα), peroxisome proliferator activated receptor γ (PPARγ), lipoprotein lipase (LPL), and osteocalcin – were found to be significantly higher than those of undifferentiated cells (Figure 4B). However, significant expression of collage type I and collagen type II was not observed in differentiated cells. These results indicate that bovine tongue epithelium-derived cells possess MSC plasticity.

## Discussion

Over the past 20 years, research on the biology of stem cells has received immense attention. A significant increase has occurred in the understanding of the characteristics of stem cells and their potentials for application in various areas [20,21]. Stem cells can engage in prolonged self-renewal and differentiation into mature cells of various lineages, which allows them to be vital cell sources for tissue engineering applications. Their remarkable ability to complement and differentiate *in vivo* is regulated by both intrinsic and extrinsic cellular mechanisms. Among the most widely used cellular types, MSCs have attracted much attention. These cells possess the ability to modulate the immune system, activate homing factors, and allow cells to access sites of injury more favorably, thus aiding in the process of tissue repair. In the veterinary field, MSCs (that were isolated from bone marrow or adipose tissue) through minimal manipulation have been applied to treat injuries of tendons, ligaments, and joint disease, with significant clinical relevance in horses and dogs under orthopedic conditions [22].

Bovine is an important agricultural species. Bovine MSCs that are derived from bone marrow and cord blood have been extensively studied. However, studies on liver-derived MSCs are rare scarce. Studies have shown that the stem cell pool in liver and liver stem cells can be divided into either non-liver-derived or liver stem cells and liver-derived liver stem cells based on differential origins [23]. Various sources of liver stem cells exhibit differences in morphology, surface markers, and differentiation. However, all encompass characteristics of multilineage differentiation potential [24,25]. Lu et al. [8] have reported the isolation and characterization of MSCs that were derived from a fetal bovine liver (LMSCs) in their study. These bovine LMSCs could be subcultured for up to 44 passages, and expressed CD29, CD44, CD73, CD90, CD106, and CD166 but not CD34, CD45, and BLA-DR. Further, bovine LMSCs exhibited substantial proliferating ability and mesodermal differentiation potential. Amniotic fluid-derived MSCs (AF-MSCs) are known to express embryonic stem cells markers such as Oct3/4, Nanog, Sox2, and SSEA3/4 as well as mesenchymal markers [26–28]. Henceforth, these cells are considered an intermediate stage between embryonic and adult stem cells. Their remarkable features allow them to serve as suitable candidates for clinical applications. Rossi et al. [1] have demonstrated that bovine AF-MSCs expressed mesenchymal markers (CD44, CD90, and CD105) and that the multilineage differentiation into mesenchymal lineages and average DT were comparable with the DT of AF-MSCs of other species [29–31]. The umbilical cord blood represents the main source of adult stem cells, which includes hematopoietic and MSCs. Raoufi et al. [7] investigated bovine umbilical cord blood-derived MSCs (UCB-MSCs) in their study and were the first to report the isolation, culture, characterization, and differentiation processes of bovine umbilical stem cells. In their study, cells from the bovine UCB were found to proliferate extensively *in vitro* and maintain their morphological and growth characteristics. Further, after several passages, these cells exhibited the same morphology and phenotype as the bovine bone marrow MSCs (BM-MSCs). Amniotic fluid, which is mainly composed of water, is the protective liquid layer that surrounds the fetus during its development. The production of amniotic fluid is determined by the excretion of fetal urine and oral, nasal, tracheal, and pulmonary fluids. Hence, the overall composition of amniotic fluid varies with gestational age. Within the Wharton’s jelly layer, MSCs have been isolated from three relatively indistinct regions: the perivascular zone, the intravascular zone, and the subamnion area. However, whether MSCs that were isolated from different compartments of the UC represent different populations remains unclear [32]. In 2006, Wharton’s jelly was – for the first time in veterinary medicine – obtained from a porcine umbilical cord [33]. In a study by Cardoso et al. [34], the bovine-derived umbilical cord-based Wharton’s jelly cells were isolated, characterized, and maintained in a three-dimensional system as an alternative source of stem cells. Endometrial tissue is a highly regenerative tissue that contains tremendously dynamic endometrial stromal cells with the capacity for growth and differentiation during the estrous cycle and pregnancy duration in cows [35]. The presence of endometrial MSCs has been described in other mammals, such as humans, pigs, ovines, and mice. Recently, Moraes et al. [10] were first to report that bovine endometrial tissue-derived MSCs (eMSCs) can be used as a new source of MSCs. Bovine eMSCs derived that are from estral uteri can adhere to plastic with fibroblastoid morphology, differentiation potentials, and immunophenotypic progenitor/stem cells characteristics, besides having an excellent viability rate after thawing.

As of now, the establishment of boT-MSCs has not yet been reported. The present study aimed to establish the culture conditions of boT-MSCs and fully explore their biological characteristics and differentiation potentials in Korean native cattle. An important feature of stem cells is its self-renewal and differentiation capacity. The present study revealed that it was possible to culture boT-MSCs *in vitro* and passage such cells for at least 20 passages using low-glucose DMEM. Yang et al. [36] have reported that varying the culture medium and passage can affect the growth characteristics, surface marker distributions, and differentiation potentials of human BM-MSCs. Thus, selecting an expansion medium can significantly influence the growth, differentiation potential, and surface marker expression of MSCs. Low-glucose DMEM is known to be a medium that is commonly used for stem cell culture [6–8,15]. DMEM and Ham’s F12 medium at 1:1 have been used to isolate and culture mouse tongue-derived endodermal stem/progenitor cells [12]. IMDM and Ham’s F12 medium at a ratio of 1:1 have also been used to isolate bovine fetal epithelium cells and fetal goat tongue cell lines [13,14]. In the present study, we isolated MSCs from bovine tongue tissue and characterized boT-MSCs by analyzing cell growth curves, cell surface markers, and differentiation potentials in three different culture media: low-glucose DMEM, low-glucose DMEM and Ham’s F12 medium at 1:1, and IMDM and Ham’s F12 medium at 1:1. We observed the superiority of using DMEM to proliferate and maintain characteristics by continuous cell passaging. These results indicate that the cultured boT-MSCs possessed quality cell proliferating ability under DMEM culture conditions.

It has been reported that bovine adipose tissue, bone marrow, liver, skin, amniotic fluid, and endometrium-derived MSCs positively express CD13, CD29, CD44, CD73, CD90, and CD105, but negatively express CD34 and CD45 [8,9,37–41]. In our study, cells were positively expressed for CD13, CD29, CD44, CD73, CD90, CD105, CD166, and MHC-I, but negative for CD14, CD31, CD34, CD45, CD106, CD117, and MHC-II. In terms of previous findings, Corradetti et al. [37] have reported that CD14 is not expressed in bovine AF-MSCs. Kato et al. have reported that CD31 is not expressed in bovine BM-MSCs [38] and Sun et al. [39] have reported that CD106 is not expressed in bovine dermal-derived MSCs. On the other hand, Lu et al. [8] have reported that CD106 is expressed in bovine LMSCs. Ren et al. [40] have reported that CD13 is indeed expressed in bovine adipose tissue-derived MSCs. CD117 also has differential expression based on tissue type. For example, bovine eMSCs express CD117, whereas bovine BM-MSCs do not express CD117 [38,41]. It is also well known that CD90 is a strong MSCs marker. Moraes et al. [42] have reported that reduced CD90 expression enhances the osteogenic and adipogenic differentiation of MSCs *in vitro*. We have conducted research in the laboratory for 10 years to establish 245 MSCs from a total of 11 species (equine, canine, feline, porcine, caprine etc) and 13 tissues (adipose tissue, skin, bone marrow, lung, umbilical cord etc). Further, the characteristics of MSCs were found to differ slightly between tissues (data not shown).

Another defining characteristic of MSCs is their multipotent capability [43,44]. In the presence of established lineage-specific differentiation factors, we demonstrated that boT-MSCs exhibited the ability to differentiate *in vitro* into mesodermal cell lineages, such as adipocytes, osteocytes, and chondrocytes. As a result of mesodermal differentiation, we confirmed mesodermal cell lineages-specific staining and expression of differentiation potential markers in differentiated cells. However, collagen type I and collagen type II genes were not significantly expressed in differentiated cells. We also observed that the expression levels of the stemness markers Sox2 and Oct3/4 were higher in cells that were grown in a low-glucose DMEM culture. The expression levels of the stemness markers were maintained at late passage (P20) compared with an early passage (P3) (data not shown).

Both common and rare diseases affect the tongue, such as vascular and lymphatic lesions (infantile hemangiomas and oral varices), reactive and inflammatory processes (hairy tongue, pigmented fungiform papillae of the tongue, benign migratory glossitis, and fissured tongue), infections (oral hairy leukoplakia, herpes simplex and varicella-zoster virus infections, human papillomavirus, and candidiasis), premalignant lesions (leukoplakia and erythroplakia), malignant lesions (squamous cell carcinoma, Kaposi sarcoma, and lymphoproliferative diseases), and signs of systemic disease (nutritional deficiency and systemic amyloidosis) [45]. In particular, tongue-related diseases such as foot-and-mouth disease virus (FMDV) and bluetongue virus, are known to greatly damage the cattle. The FMDV capsid is composed of 60 icosahedral units, each of which comprises one copy of the VP1, VP2, VP3, and VP4 proteins. The VP1 protein coat contains the main antigenic determinants of the virion. Hence changes in its sequence must be responsible for the high antigenic variability of the virus. Wang et al. have screened shRNA that targets viral VP1 genes and confirmed its antiviral function in primary tongue epithelium cells from transgenic fetuses that express shRNA [13]. Li et al. have successfully produced transgenic goats that highly express 3D-7414siRNA-targeting 3D pol genes of FMDV genome. Subsequent experiments have supported the finding that tongue epithelium cells from transgenic goats effectively inhibit virus replication [46].

MSCs have been harvested from nearly all body tissues of various species [47]. Among all domesticated species, bovine has crucial importance in the economics of the livestock industry. Many researchers have established bovine MSCs from various tissues and examined the appropriate culture conditions necessary [48]. House et al. evaluated techniques to demonstrate FMDV in bovine tongue epithelium, and compared sensitivities among cell lines [49]. In the present study, we cautiously established boT-MSCs with the hopes to more effectively perform FMDV-related research in the future. As such, our research group intends to use for FMDV-related studies in the future.

## Abbreviations

AF-MSC: amniotic fluid-derived mesenchymal stem cell
BM-MSC: bone marrow mesenchymal stem cell
boT-MSC: bovine tongue tissue epithelium-derived mesenchymal stem cell
CFU-F: fibroblastic colony-forming unit
CPDL: cumulative population doubling level
CT: culture time
DMEM: Dulbecco’s modified Eagle’s medium
DT: doubling time
eMSC: endometrial tissue-derived mesenchymal stem cell
FITC: fluorescein isothiocyanate
FMDV: foot-and-mouth disease virus
IMDM: Iscove’s Dulbecco’s modified Eagle’s medium
MHC: major histocompatibility complex
MSC: mesenchymal stem cell
Oct3/4: octamer-binding transcription factor 3/4
PBS: phosphate-buffered saline
PE: phycoerythrin
PPARγ: peroxisome proliferator activated receptor γ
qRT-PCR: quantitative real-time RT-PCR
Sox2: sex determining region Y-box2
TGFβ-3: transforming growth factor β-3
